# Peptidoglycan-inspired autonomous ultrafast self-healing bio-friendly elastomers for bio-integrated electronics

**DOI:** 10.1093/nsr/nwaa154

**Published:** 2020-07-06

**Authors:** Luzhi Zhang, Jiahui Liang, Chenyu Jiang, Zenghe Liu, Lijie Sun, Shuo Chen, Huixia Xuan, Dong Lei, Qingbao Guan, Xiaofeng Ye, Zhengwei You

**Affiliations:** State Key Laboratory for Modification of Chemical Fibers and Polymer Materials, Shanghai Belt and Road Joint Laboratory of Advanced Fiber and Low-dimension Materials, College of Materials Science and Engineering, Donghua University, Shanghai 201620, China; State Key Laboratory for Modification of Chemical Fibers and Polymer Materials, Shanghai Belt and Road Joint Laboratory of Advanced Fiber and Low-dimension Materials, College of Materials Science and Engineering, Donghua University, Shanghai 201620, China; Department of Cardiac Surgery, Ruijin Hospital, Shanghai Jiao Tong University School of Medicine, Shanghai 200025, China; State Key Laboratory for Modification of Chemical Fibers and Polymer Materials, Shanghai Belt and Road Joint Laboratory of Advanced Fiber and Low-dimension Materials, College of Materials Science and Engineering, Donghua University, Shanghai 201620, China; State Key Laboratory for Modification of Chemical Fibers and Polymer Materials, Shanghai Belt and Road Joint Laboratory of Advanced Fiber and Low-dimension Materials, College of Materials Science and Engineering, Donghua University, Shanghai 201620, China; State Key Laboratory for Modification of Chemical Fibers and Polymer Materials, Shanghai Belt and Road Joint Laboratory of Advanced Fiber and Low-dimension Materials, College of Materials Science and Engineering, Donghua University, Shanghai 201620, China; State Key Laboratory for Modification of Chemical Fibers and Polymer Materials, Shanghai Belt and Road Joint Laboratory of Advanced Fiber and Low-dimension Materials, College of Materials Science and Engineering, Donghua University, Shanghai 201620, China; State Key Laboratory for Modification of Chemical Fibers and Polymer Materials, Shanghai Belt and Road Joint Laboratory of Advanced Fiber and Low-dimension Materials, College of Materials Science and Engineering, Donghua University, Shanghai 201620, China; State Key Laboratory for Modification of Chemical Fibers and Polymer Materials, Shanghai Belt and Road Joint Laboratory of Advanced Fiber and Low-dimension Materials, College of Materials Science and Engineering, Donghua University, Shanghai 201620, China; Department of Cardiac Surgery, Ruijin Hospital, Shanghai Jiao Tong University School of Medicine, Shanghai 200025, China; State Key Laboratory for Modification of Chemical Fibers and Polymer Materials, Shanghai Belt and Road Joint Laboratory of Advanced Fiber and Low-dimension Materials, College of Materials Science and Engineering, Donghua University, Shanghai 201620, China

**Keywords:** biomimetic, elastomers, ultrafast self-healing, bio-integrated electronics, stretchable conductor

## Abstract

Elastomers are essential for stretchable electronics, which have become more and more important in bio-integrated devices. To ensure high compliance with the application environment, elastomers are expected to resist, and even self-repair, mechanical damage, while being friendly to the human body. Herein, inspired by peptidoglycan, we designed the first room-temperature autonomous self-healing biodegradable and biocompatible elastomers, poly(sebacoyl 1,6-hexamethylenedicarbamate diglyceride) (PSeHCD) elastomers. The unique structure including alternating ester-urethane moieties and bionic hybrid crosslinking endowed PSeHCD elastomers superior properties including ultrafast self-healing, tunable biomimetic mechanical properties, facile reprocessability, as well as good biocompatibility and biodegradability. The potential of the PSeHCD elastomers was demonstrated as a super-fast self-healing stretchable conductor (21 s) and motion sensor (2 min). This work provides a new design and synthetic principle of elastomers for applications in bio-integrated electronics.

## Teaser

Superfast autonomous self-healing biocompatible and biodegradable elastomers were developed based on unique alternating ester-urethane moieties and bionic hybrid crosslinking networks, showing great potential for stretchable electronics.

## INTRODUCTION

In recent years, there has been rapid development in bio-integrated electronics including electronic skins, wearable electronics and implantable electronics [1–3]. Current electronics usually use brittle and rigid substrates, which cause mechanical mismatch with soft and frequently deformed tissues [4]. Stretchable bio-integrated electronics based on elastomers have shown great promise to address such challenges [5–7]. However, because of the dynamic application environment, elastomers are subjected to continuous mechanical loading and may suffer from mechanical failure and microcracks. One potential way to address this problem is endowing a self-healing property on elastomers [8–12]. The healing process of most self-healing elastomers requires external stimuli such as heat and light, which can be unfriendly to the human body [13–16]. Furthermore, self-healing elastomers usually have slow healing speed (typically taking hours to days), which may cause long-term failure of electronics [17–20]. Rapid autonomous self-healing elastomers are highly desirable, but remain a challenge. Furthermore, as bio-integrated electronics are directly in contact with human tissues, the biological design including biocompatibility and biodegradability of self-healing elastomers requires special attention, but has barely been investigated.

**Figure 1.**
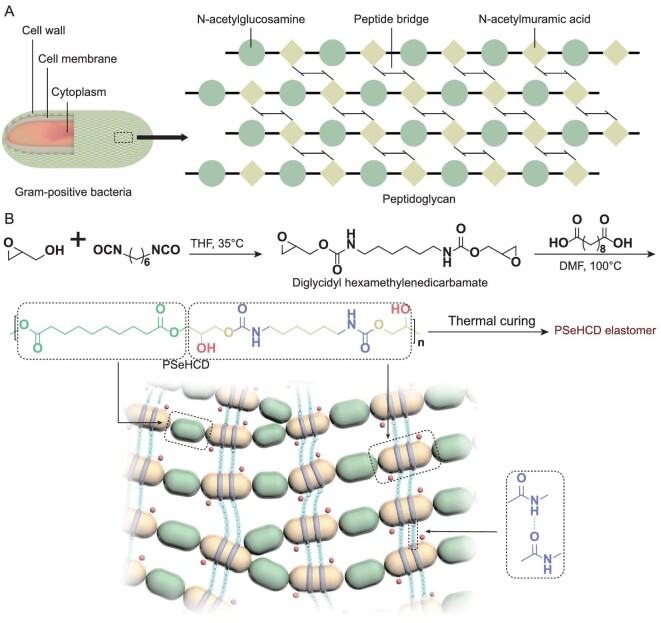
Design and synthesis of PSeHCD elastomers. (A) Schematic of peptidoglycan of Gram-positive bacteria. (B) Schematic of the synthesis and structure of PSeHCD elastomers with both extensive evenly distributed H-bonds physical crosslinking and controlled partially chemical crosslinking.

Herein, we set out to design a nature-inspired structure to address the above challenges in elastomers for bio-integrated electronics. Peptidoglycan is the major component of most bacterial cell walls and plays a crucial structural role in maintaining the mechanical strength and integrity of the cell wall [21]. The function of peptidoglycan derives from the unique three-dimensional networks constructed by the backbone of the alternating heteropolysaccharide (*N*-acetylglucosamine and *N*-acetylmuramic acid) chain and the extensive evenly distributed crosslinked peptide side chain (Fig. 1A). Accordingly, we designed a peptidoglycan-mimicking alternating polyester-*co*-polyurethane elastomer, poly(sebacoyl 1,6-hexamethylenedicarbamate diglyceride) (PSeHCD) elastomer, with chemically and physically hybrid crosslinking (Fig. 1B). To the best of our knowledge, this is the first report on autonomous self-healing biocompatible and biodegradable elastomers. The key to synthesize PSeHCD is our previously developed acid-induced epoxide ring-opening polymerization [22]. PSeHCD was designed according to the following considerations. Each repeating unit had two parts: ester and urethane groups. The alternating ester groups ensured steady degradation behavior. The alternating urethane groups introduced extensive and evenly distributed hydrogen bonds (H-bonds), which made the physically crosslinked dynamic networks strong and the self-healing efficient. The pendent hydroxyl groups of PSeHCD benefit from integration with tissues, and enable facile post-functionalization including self-esterification with terminal carboxylic acid groups to enhance its molecular weight and, consequently, mechanical strength. Because of the regular structure, the terminal carboxylic acid groups of PSeHCD are limited, which ensure low crosslinking density to guarantee macroscopic self-healability and thermoplasticity. Furthermore, hexamethylenedicarbamate and glycerol sebacate, proven to be biocompatible, were selected as the building blocks to ensure good biocompatibility of resultant elastomers [23,24].

## RESULTS AND DISCUSSION

### Preparation and characterization of PSeHCD and resultant elastomers

The synthesis of PSeHCD included two steps (Fig. 1B). The first was preparation of the monomer diglycidyl hexamethylenedicarbamate. Considering the high reactivity of the isocyanate and epoxide groups, the reaction was performed under an anhydrous environment without catalyst. The second step was acid-induced epoxide ring-opening polymerization between equimolar amounts of sebacic acid and diglycidyl hexamethylenedicarbamate in the presence of catalyst *n-*Bu_4_NBr [22]. The resultant PSeHCD had a moderate molecular weight (*M_n_* = 15.5 kDa, *M_w_* = 25.2 kDa) and a low polydispersity index of 1.62. Nuclear magnetic resonance (NMR) and Fourier transformed infrared spectroscopy (FTIR) were used for structural characterization. In the ^1^H NMR spectrum of the monomer (Fig. S1A), the signals marked ‘a’, ‘b’, ‘c’, ‘d’ and ‘e’ at chemical shift δ 2.75, 2.59, 3.14, 4.36 and 3.68 ppm corresponded to the CH_2_ and CH protons of the glycidol moiety. The signals marked ‘g’ and ‘h’ at chemical shift δ 2.94 and 1.24–1.36 corresponded to the CH_2_ of the hexamethylene diisocyanate (HDI) moiety. The signals of -NH- group marked ‘f’ appeared at chemical shift δ 7.27 ppm. In the ^1^H NMR spectrum of PSeHCD (Fig. S1B), the signals marked ‘a’, ‘h’ and ‘b, i’ at chemical shift δ 2.26, 2.94 and 1.25–1.5 corresponded to the CH_2_ of the sebacoyl and HDI moiety in the polymer backbone. The signals marked ‘c, d’ and ‘e’ at chemical shift δ 3.76–4.38 and 4.77 corresponded to the CH_2_ and CH protons of the glycidol moiety. The signals marked ‘f’ and ‘g’ at chemical shift δ 5.75 and 7.74 corresponded to the -OH and -NH group. Moreover, the integrations of ‘a’ and ‘h’ were essentially identical, which revealed that equimolar amounts of sebacic acid and diglycidyl hexamethylenedicarbamate reacted. The FTIR spectrum of PSeHCD (Fig. S1C) clearly displayed the characteristic signal of hydroxyl groups in the side chain (the intense stretching vibration of O-H bonds at 3439 cm^−1^), which was absent from the spectrum of its monomer. The peaks corresponding to the stretching vibrations of C=O and N-H groups at 1600–1700 cm^−1^ and 3300–3500 cm^−1^ were similar in polymer and monomer, confirming the presence of a urethane bond. Moreover, no signals associated with -NCO groups (around 2270 cm^−1^) were observed, indicating the complete consumption of the isocyanate groups. The resultant PSeHCD had a unique alternating ester-urethane structure, which is key for its mechanical and self-healing properties. The polymerization is versatile and applicable to different isocyanates, epoxides and polyacids, and could produce a family of sophisticated hybrid materials.

It is difficult to obtain high molecular weight heterochain polymers with functional groups, as this usually requires tedious synthesis or harsh reaction conditions [25,26]. Unlike conventional polycondensation reactions, the acid-induced epoxide ring-opening polymerization used to fabricate PSeHCD can produce polymer chains with extensive pendent hydroxyl groups during polymerization without typical careful protection and deprotection steps. The self-esterification between hydroxyl groups and terminal carboxylic acid groups significantly enhanced the molecular weight and consequent mechanical strength of the PSeHCD elastomers. The mechanical properties of PSeHCD elastomers can be easily adjusted by controlling the curing time. Although PSeHCD elastomers may be partially crosslinked, the sparse terminal carboxylic acid groups in PSeHCD elastomers guaranteed low crosslinking density to ensure macroscopic thermoplasticity while significantly increasing the molecular weight of the microscopic structure. This strategy is simple and versatile, and could be extended to obtain other high molecular weight functional heterochain polymers. For comparison, PSeHCD was cured with additional sebacic acid under conditions identical to the previously reported sebacic acid crosslinked poly(sebacoyl diglyceride) (PSeD-SA) elastomer to produce fully chemically crosslinked PSeHCD-SA elastomer [27].

Physical properties including the solubility, hydrophilicity and thermal properties of PSeHCD and resultant elastomers were investigated. PSeHCD exhibited good solubility in common polar organic solvents (Table S1), which made for easy processing and modification. According to the solubility test (Table S2), the increased ratios of insoluble fractions of PSeHCD elastomers with increasing curing time indicated increased crosslinking density. The insoluble fraction of PSeHCD-72 and PSeHCD-96 varied little likely because of the limited availability of terminal carboxylic acid groups for crosslinking, which was almost consumed within 72 h.

The hydrophilicity was obtained by measuring the water contact angle of polymer films (Fig. S2A). The static air-water contact angles of PSeHCD, PSeHCD-48, PSeHCD-60 and PSeHCD-72 elastomers were 66.6° ± 2.4°, 66.7° ± 2.7°, 67.6° ± 2.7° and 68.4° ± 2.6°, respectively, which are in the favorable range for cell growth and adhesion. PSeHCD-SA elastomer had relatively higher water contact angle (86.1° ± 1.3°), likely because of the significant consumption of hydroxyl groups during the curing process.

Differential scanning calorimetry (DSC) results revealed that PSeHCD had a low glass transition temperature (*T*_g_) of −15°C (Fig. S2B). Self-crosslinked PSeHCD elastomers showed a gradually increased *T*_g_ (−14.8°C for PSeHCD-48, −9.8°C for PSeHCD-60 and −5.9°C for PSeHCD-72) with increase in degree of crosslinking. PSeHCD-SA had the highest *T*_g_ of 9.8°C among all PSeHCD elastomers because it had the highest crosslinking density. The *T*_g_s of PSeHCD elastomers were all below room temperature and no crystallization was observed from −50°C to 150°C. The totally amorphous structure ensured good elasticity and chain mobility, beneficial in terms of rapid self-healing properties. Thermal gravimetric analysis (TGA) (Fig. S2C) showed that both PSeHCD and resultant elastomers had a high decomposition temperature (over 200°C). Overall, PSeHCD and the resultant elastomers can be used in a relatively wide temperature range.

### Mechanical properties of PSeHCD elastomers

**Figure 2.**
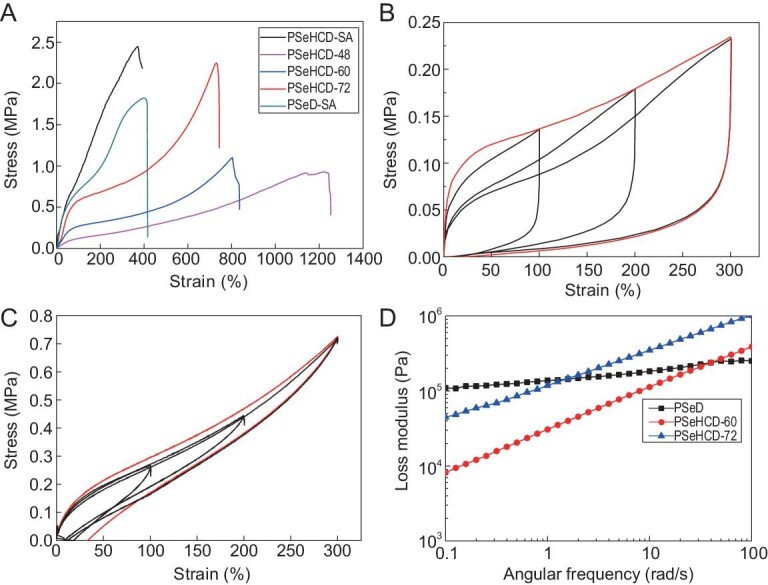
Mechanical properties of PSeHCD and PSeD elastomers. (A) Typical stress-strain curves of PSeHCD-SA, PSeHCD-48, PSeHCD-60, PSeHCD-72 and PSeD-SA elastomers. Representative data of PSeD-SA in our previous report were used for comparison [27]. A single cyclic tensile to 300% (red line) and three subsequent cyclic tensile (black line) of (B) PSeHCD-60 and (C) PSeHCD-72. (D) Rheology curves (loss modulus versus angular frequency) for PSeHCD-60, PSeHCD-72 and PSeD elastomers.

The mechanical properties of self-crosslinked PSeHCD elastomers were investigated via tensile tests using fully chemically crosslinked PSeHCD-SA and PSeD-SA as control groups (Fig. 2A). PSeHCD-SA showed a relatively high Young's modulus of 4.3 ± 0.28 MPa and, consequently, a deduced high crosslinking density of 578.5 ± 37.6 mol m^−3^ by covalent bonds, which is in accordance with the aforementioned solubility test (Table S2). Furthermore, PSeHCD-SA showed higher tensile strength and toughness, while the elongation at break was kept at the same level, compared to PSeD-SA (Fig. 2A, Table 1). The improved mechanical properties could be attributed to extensive evenly distributed H-bonds formed by the urethane moiety in PSeHCD-SA, which was absent from PSeD-SA.

**Table 1.** Mechanical properties of PSeHCD elastomers and PSeD-SA elastomer.

Sample	Tensile strength (MPa)	Young's modulus (MPa)	Elongation at break (%)	Crosslinking density (mol m^−3^)	Toughness (MJ m^−3^)
					
PSeHCD-SA	2.43 ± 0.18	2.39 ± 0.28	408 ± 66	321.55 ± 37.67	5.53 ± 0.24
PSeHCD-48	0.96 ± 0.05	0.15 ± 0.02	1109 ± 159	20.45 ± 2.83	5.46 ± 0.20
PSeHCD-60	0.97 ± 0.04	0.41 ± 0.05	818 ± 53	54.75 ± 6.73	4.59 ± 0.57
PSeHCD-72	2.26 ± 0.13	0.80 ± 0.04	770 ± 26	107.90 ± 5.38	8.31 ± 1.44
PSeD-SA	1.57 ± 0.48	1.83 ± 0.06	409 ± 29	250.24 ± 8.07	4.46 ± 0.97

Self-crosslinked PSeHCD elastomers showed significantly larger elongation at break than fully chemically crosslinked PSeHCD-SA elastomer. The tensile strength increased with crosslinking density ranging from 0.96 ± 0.05 MPa to 2.26 ± 0.13 MPa, comparable to PSeHCD-SA. PSeHCD-72 exhibited the highest toughness of 8.31 ± 1.44 MJ m^−3^, 1.5 times that of PSeHCD-SA, likely because of the efficient energy dissipation of sacrificial H-bonds in the hybrid crosslinked system. In the process of stress, a large number of uniformly distributed H-bonds were gradually broken to dissipate energy without sudden release of energy. As a result, the Young's moduli of PSeHCD elastomers was kept at a relatively low value, in the range of that of soft tissues. Compared to the previously reported similar elastomers poly(glycerol sebacate) (PGS) and poly (glycerol sebacate urethane) (PGSU-SF 1 : 0.3), PSeHCD elastomers showed superior mechanical properties at enhancing maximum elongation (770 ± 26% for PSeHCD-72, compared to 200 ± 30% for PGS and 121 ± 27% for PGSU-SF 1 : 0.3) and tensile strength (2.26 ± 0.13 MPa for PSeHCD-72, compared to 0.38 ± 0.06 MPa for PGS and 1.00 ± 0.29 MPa for PGSU-SF 1 : 0.3) [28]. These results indicated that a suitable combination of extensively evenly distributed H-bonds and controlled partially chemical crosslinking would provide a synergistic effect for optimized mechanical performance.

To verify the effect of sacrificial bonds in the self-crosslinked elastomers, PSeHCD-60 (Fig. 2B) and PSeHCD-72 (Fig. 2C) elastomers were subjected to three consecutive loading–unloading cycles without any intervals. Both showed significant hysteresis during the first cycle confirming large energy dissipation of sacrificial H-bonds. PSeHCD-72 showed quick recovery without any apparent drop in tensile stress and Young's modulus during three consecutive loading–unloading cycles likely because of its relatively high chemical crosslinking density, which held the molecular chains closer and facilitated reformation of H-bonds.

The mechanical performance of self-crosslinked PSeHCD elastomers was further evaluated by dynamic mechanical analysis (DMA) and rheology measurements [29]. A DMA varying temperature test from 30°C to 100°C of PSeHCD-60 (Fig. S3) showed that the storage modulus G′ was always higher than the loss modulus G′, indicating stable 3D networks of PSeHCD-60. A frequency sweeping rheology test showed that the loss moduli of PSeHCD-60 and PSeHCD-72 displayed a clear upward trend with increasing frequency, while that of PSeD remained almost consistent (Fig. 2D). These results were in accordance with previous studies of sacrificial H-bonds. When the experimental frequencies were much lower than H-bond kinetics (equivalent to high temperature), the dissociation of H-bonds was fast leading to significant dissipation of energy. When the frequencies increased (equivalent to low temperature), the sacrificial H-bonds and subsequent chain relaxation was limited, resulting in an apparent increase of loss modulus. As the chemically crosslinked PSeD elastomer had negligible H-bonds, the loss modulus under shear stress mainly depended on covalent crosslinking leading to a slightly increased loss modulus.

### Self-healing and reprocessing of PSeHCD elastomer

**Figure 3.**
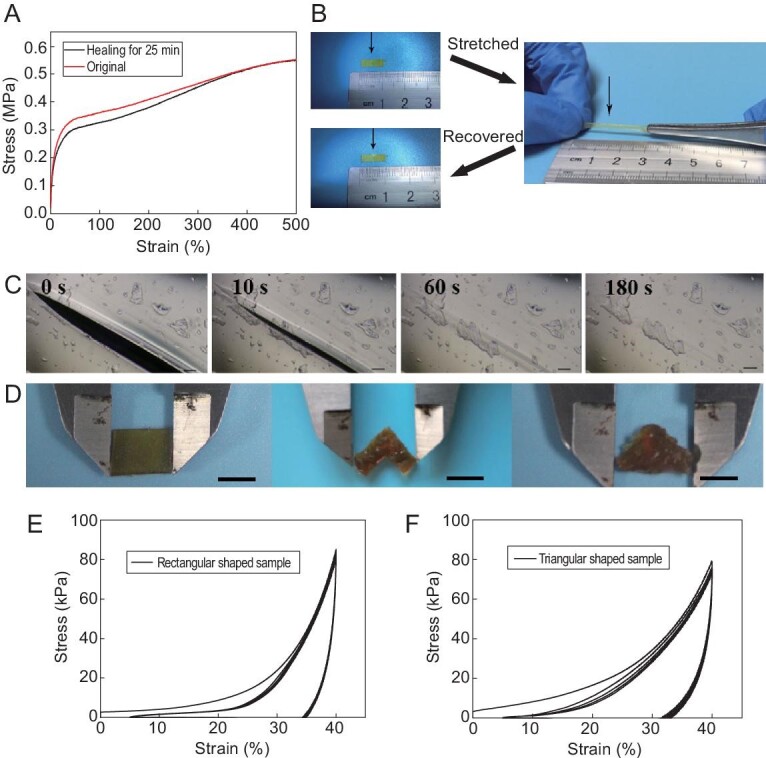
Self-healing and reprocessing of PSeHCD elastomers. (A) Stress-strain curves of original and self-healed PSeHCD-60 strips. (B) Optical images of the healed PSeHCD-60 strip before and after stretching; the black arrow referred to the healed area. (C) Microscopic images of self-healing procedure of a scratch on the surface of a PSeHCD-60 film. The scratch completely healed within 3 min at 15°C (scale bars: 50 μm). (D) Reprocessing and reshaping of PSeHCD-72 at 100°C for 10 min (scale bars: 5 mm). Cyclic compression stress-strain curves of (E) original (rectangular shaped) and (F) reprocessed (triangular shaped) PSeHCD-72 elastomer verified the retention of the good elasticity after being reprocessed.

Self-healing would significantly expand the service life of materials, and therefore would be highly desirable for economic and safe utilization. Unlike conventional covalently crosslinked elastomers, PSeHCD elastomers possess excellent self-healing ability. PSeHCD-60 was taken as a representative to demonstrate the self-healing of PSeHCD elastomers. First, a scratch healing test was performed on PSeHCD-60 film. A scratch of 100 μm healed almost immediately and completely disappeared within 3 min at 15°C without any external stimuli (Fig. 3C). Then, the PSeHCD-60 strip was cut into two pieces, which were slightly rejoined and placed at room temperature for 25 min. The self-healed strip showed a similar stress-strain curve to the original (Fig. 3A and B), and comparable toughness (4.05 MJ m^−3^) at over 90% of the original (4.42 MJ m^−3^).

The key to realize efficient self-healing of PSeHCD elastomers was the extensive evenly distributed H-bonds. The self-healing ability of material provided by a single H-bond was weak. ‘Many a little makes a mickle.’ Furthermore, regular distribution of H-bonds in the unique alternating structure greatly enhanced the probability and speed of H-bond reformation at the damage area. The relatively low *T*_g_ (−9.8°C for PSeHCD-60) resulted in high mobility of the molecular chain at ambient temperature, which is beneficial to self-healing. If the individual H-bonds were too strong, such as the reported multiple H-bonds, the polymeric networks were restricted and the self-healing ability was reduced [30]. On the other hand, the degree of chemical crosslinking was important. Partial chemical crosslinking essentially increased the molecular weight of individual units, leading to a significant increase in H-bonds between them to furnish the stable networks. At the same time, controlled chemical crosslinking ensured the thermoplasticity. The reprocessing of PSeHCD elastomers was performed at 100°C. As an example, PSeHCD-72 elastomer was readily thermally processed from a rectangle to a polygon, then to a triangle (Fig. 3D). This demonstrated that the H-bonds in the networks could dissociate upon heating and the covalent crosslinked networks were controlled on a microscopic scale. Furthermore, the original rectangular sample and final triangular sample exhibited a similar good elasticity according to cyclic compression tests (Fig. 3E and F). This revealed that the H-bonds could readily reform upon cooling and the chemical crosslinking was stable during processing, resulting in a reliable hybrid crosslinked network and mechanical performance.

### Biocompatibility, degradability and functionalization of PSeHCD elastomers

**Figure 4.**
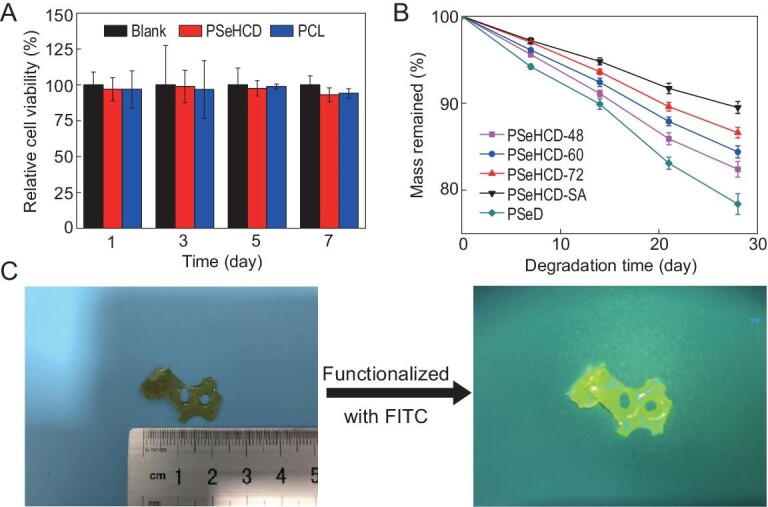
*In vitro* biocompatibility, degradability and functionalization of PSeHCD elastomers. (A) CCK-8 assay of fibroblasts cultured on PSeHCD elastomer and PCL for 1, 3, 5 and 7 days. The cell viability on PSeHCD elastomer and PCL groups were compared with blank group to calculate relative cell viability. There was no significant difference between PSeHCD and PCL at the same time point. (B) *In vitro* degradation of PSeD and PSeHCD elastomers in PBS solution for 4 weeks at 37°C. (C) Functionalization of PSeHCD. Optical pictures of a pony-shaped sample of PSeHCD-72 elastomer and the fluorescent image after being chemically functionalized by FITC.

PSeHCD elastomers are designed as human- and environmentally friendly materials, the biocompatibility and degradability of the PSeHCD elastomers were investigated. Fibroblasts were seeded on PSeHCD elastomer to evaluate the *in vitro* biocompatibility using polycaprolactone (PCL) as a positive control, which is main component in numerous medical devices approved by the FDA. A Cell Counting Kit-8 (CCK-8) assay revealed that there was no significant difference between the PSeHCD elastomers and the control within 7 days (Fig. 4A). These results demonstrate the good cytocompatibility of PSeHCD elastomers, suitable for biomedical applications.

Polyester is the most investigated biodegradable materials, while polyurethane is usually hard to degrade especially by hydrolysis. Accordingly, we designed PSeHCD with an alternating structure of ester and urethane, to ensure good degradability of the PSeHCD elastomers. As expected, all PSeHCD elastomers degraded steadily in phosphate buffered saline (PBS, pH = 7.2–7.4). The degradation rate corresponded well with the degree of crosslinking (Fig. 4B). PSeHCD-SA, with the highest crosslinking density, showed the lowest degradation rate. PSeHCD-48, with the lowest crosslinking density, showed the highest degradation rate, but this was still lower than that of PSeD without urethane units. These results indicate that the degradability of PSeHCD elastomers can be tuned across a wide range by regulating the content of urethane units or crosslinking degree for specific applications.

As designed, the extensive pendent hydroxyl groups in PSeHCD enable facile modifications of PSeHCD including functionalization, which is of great significance for numerous applications. As a proof of principle, fluorescein isothiocyanate (FITC) was readily bonded to PSeHCD-72 to produce a yellowish-green fluorescent pony-shaped sample (Fig. 4C).

### Application of the PSeHCD elastomer in bio-integrated electronics

**Figure 5.**
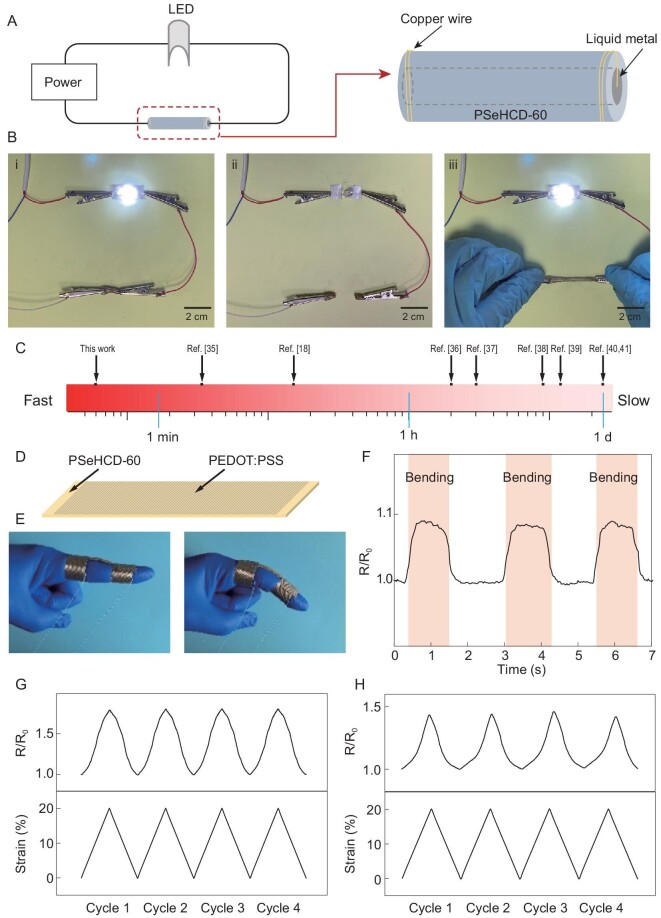
Superfast self-healing and stretchable conductor and sensor. (A) Schematics of the simple electronic circuit and the conductor. (B) Demonstration of the superfast self-healable and stretchable properties. The LED was lit at the original stage (i), and turned off after the conductor being cut (ii). The severed conductor was contacted just 21 s under ambient conditions and kept the LED on even while being stretched to 340% (iii). (C) Comparison of healing time among our work and other room temperature self-healing and stretchable conductors reported in the literature [18,35–41]. (D) Schematic of the sensor. (E) Photographs showing the monitoring experiment of finger bending. (F) Dynamic response of electrical resistance (R/R_0_) of sensor during finger bending. (G, H) Electrical resistance (R/R_0_) response to strain of (G) original and (H) healed (self-healing for 2 min at room temperature) sensors in loading and unloading cycles at 20% strain.

Stretchable and self-healable electronic materials are highly desirable for emerging bio-integrated electronics, but remain a great challenge. The healing process of electronics usually requires an external energy input such as heat [31,32] or laser [33,34], which are unfriendly to the human body. As a demonstration, we fabricated a stretchable and self-healable PSeHCD-60/liquid metal composite conductor and PSeHCD-60/poly(3,4-ethylenedioxythiophene): poly(styrenesulfonic acid) (PEDOT : PSS) sensor (Fig. 5).

The PSeHCD elastomer composite conductor could efficiently light up a light-emitting diode (LED) lamp in a circuit applied voltage of 3 V (Fig. 5A and B). Once the conductor was cut into two halves, the LED was off. When the two pieces contacted for 21 s at room temperature without any external energy input, the LED could be lit up again and the healed conductor could also be stretched to 340%. A PSeHCD elastomer composite conductor showed the fastest self-healing speed at room temperature of previously reported stretchable conductors (Fig. 5C) [18,35–41].

The PSeHCD elastomer composite sensor can be used to monitor bodily motions (Fig. 5D). The composite sensor was fixed on a forefinger by two conductive tapes as a wearable device to monitor the joint motion in real-time (Fig. 5E). When the forefinger was bending, an instant increase of resistance was observed (Fig. 5F). The relationship between the response of the resistance of the composite sensor and strains was also tested (Fig. 5G). Compared with recent reports, PSeHCD elastomer composite sensor exhibits higher strain sensitivity ((R/R_0_)/strain) [42–44]. Furthermore, the healed composite sensor, which was completely cut off and healed for 2 min at room temperature, exhibited a dynamic response of electrical resistance (R/R_0_) to the strain in a cyclic stretching test similar to the original intact sensor (Fig. 5H).

## CONCLUSION

In summary, we proposed and fabricated the first autonomous self-healing biocompatible and biodegradable elastomers for bio-integrated electronics. For the first time, the peptidoglycan-inspired molecular design principle has been used in synthetic polymers. The bionic PSeHCD elastomers with unique alternating ester-urethane moieties and chemically and physically hybrid crosslinking showed ultrafast autonomous self-healing, biomimetic mechanical properties and facile reprocessability, in addition to good biocompatibility and degradability. The potential applications of PSeHCD elastomers in bio-integrated electronics were demonstrated as a fast self-healing conductor and sensor. The synthetic method of PSeHCD is simple, versatile and easily scaled up. We expect the design principle and synthetic strategy in this work will inspire a series of new functional smart materials for a wide range of applications such as stretchable electronics, soft robots and biomedical engineering.

## METHODS

### Synthesis of monomer diglycidyl hexamethylenedicarbamate

Anhydrous and anaerobic treatment was performed in a glove box to maintain the reaction under a nitrogen atmosphere. HDI (4.500 mL, 28.09 mmol) was mixed dropwise with a solution of glycidol (4.128 mL, 63.69 mmol) and tetrahydrofuran (THF, 10 mL). The mixture was stirred at 35°C for 40 h. It was then purified by precipitation in ethyl ether, and vacuum-dried. The monomer diglycidyl hexamethylenedicarbamate (4.52 g, 50.8% yield) was a white flocculent solid.

### Synthesis of PSeHCD

Diglycidyl hexamethylenedicarbamate (0.3000 g, 0.9483 mmol), sebacic acid (0.1918 g, 0.9483 mol) and tetrabutylammonium bromide (2.600 mg, 0.85 mmol) were mixed and dissolved in anhydrous *N,N*-dimethylformamide (DMF, 0.5 mL) in a sealed flask in a glove box filled with nitrogen. The mixture was stirred at 60°C for half an hour, then the temperature was increased to 100°C for another 65 h. The reaction mixture was purified by dilution in ethyl acetate and precipitated in ethyl ether three times. The product was dried in a vacuum at 60°C overnight to obtain a light-yellow solid (85% yield).

### Fabrication of PSeHCD elastomer

PSeHCD and sebacic acid (1.1 wt.%) were dissolved in acetone. After removing solvent by rotary evaporation, the solvent-free mixture was melted and poured into a Teflon mold, heated at 120°C for 20 h to remove the bubbles, then subjected to a vacuum (1.1 Torr) at 120°C for another 21 h to yield crosslinked PSeHCD-SA elastomer. PSeHCD without any other additive was melted at 120°C and poured into a Teflon mold, heated at 120°C for 20 h to remove the bubbles, then subjected to a vacuum (1.1 Torr) at 120°C for another 48 h, 60 h, 72 h and 96 h to yield PSeHCD-48, PSeHCD-60, PSeHCD-72 and PSeHCD-96 elastomers, respectively.

### Characterizations

FTIR spectra were recorded on an Avatar 380 spectrometer. NMR spectra were recorded on a Bruker AM-400 in DMSO-d_6_. The molecular weight was determined *via* gel permeation chromatography (GPC) on a Malvern GPC system consisting of GPC Max with a Dual 270 triple detector array, and DMF (HPLC grade) was used as the eluent at a flow rate of 0.7 mL min^−1^ at 40°C. DSC experiments were carried out on Netzsch DSC204F1 from −50°C to 150°C at a heating rate of 10°C min^−1^ in nitrogen atmosphere. TGA were carried out on a Q5000IR instruments from ambient temperature to 500°C at a heating rate of 10°C min^−1^ in a nitrogen atmosphere. Tensile tests were conducted on an MTS insight mechanical analyzer (more details can be found in the Supplementary data). The dynamic mechanical analysis (DMA) was carried out with the frequency sweeping from 1 Hz to 100 Hz at 25°C on a TA-Q800. The rheological properties were measured by ARES-RFS rheometer (more details can be found in the Supplementary data). The calculation of crosslinking density and insoluble fraction are described in Supplementary data. The resistance change was measured with a Keithley DMM7510 system electrometer. The self-healing, reprocessing, functionalization, degradation and biocompatibility testing can be found in the Supplementary data.

## Supplementary Material

nwaa154_Supplemental_File
