# Supplementary material for: Peptidoglycan-inspired autonomous ultrafast self-healing bio-friendly elastomers for bio-integrated electronics
Source: Natl Sci Rev. 2020 Jul 6;8(5):nwaa154. doi: 10.1093/nsr/nwaa154 (PMC8288426; doi:10.1093/nsr/nwaa154)
Supplement: nwaa154_Supplemental_File [file nwaa154_supplemental_file.docx]

**SUPPLEMENTARY DATA**

**Peptidoglycan-inspired autonomous ultrafast self-healing bio-friendly elastomers for bio-integrated electronics**

Luzhi Zhang^1,^†, Jiahui Liang^1,^†, Chenyu Jiang^2^, Zenghe Liu^1^, Lijie Sun^1^, Shuo Chen^1^, Huixia Xuan^1^, Dong Lei^1^, Qingbao Guan^1^, Xiaofeng Ye^2^ and Zhengwei You^1,^*

^1^State Key Laboratory for Modification of Chemical Fibers and Polymer Materials, Shanghai Belt and Road Joint Laboratory of Advanced Fiber and Low-dimension Materials, College of Materials Science and Engineering, Donghua University, Shanghai 201620, China

^2^Department of Cardiac Surgery, Ruijin Hospital, Shanghai Jiao Tong University, School of Medicine, Shanghai 200025, China

*Corresponding author. E-mail: zyou@dhu.edu.cn

†Equally contributed to this work.

**Materials**

Sebacic acid (J&K, 98%) was recrystallized three times from 95% ethanol and dried by vacuum dryer. Tetrahydrofuran (THF, Yonghua, AR) was dried by sodium. Glycidol (J&K, 96%), hexamethylene diisocyanate (HDI, J&K, 99%), N,N-dimethylformamide (DMF, J&K, anhydrous), fluorescein isothiocyanate (FITC, J&K, 97%), poly(3,4-ethylenedioxythiophene):poly(styrenesulfonic acid) (PEDOT:PSS, J&K, CLEVIOS P VP AI 4083), ethyl ether (Yun Li, AR), ethyl acetate (Yun Li, AR), acetone (Yun Li, AR), and tetrabutylammonium bromide (*n*-Bu_4_NBr, TCI, 99%) were used without further purification. Deionized water was used in washings.

**Characterizations**

**Tensile test****:** Tensile test was conducted on an MTS insight mechanical analyzer equipped with a 50 Newton load cell according to ASTM standard D142-06a. Three dog bone-shaped samples (D142-06a die A design scaled by 1/4, 14.75 mm × 3 mm × 1.3 mm, length × width × thickness) were tested and averaged for every type of PSeHCD elastomer. In the simple tensile test, the sample was elongated to failure with a deflection speed of 125 mm min^-1^. In the single cyclic tensile test with a strain of 300%, the loading and unloading speed was 125 mm min^-1^ and 2 mm min^-1^, respectively. In the three subsequent cyclic tensile tests with a strain of 100% followed by two extensions to 200% and 300% without any interval, the loading and unloading speed was 125 mm min^-1^ and 2 mm min^-1^, respectively. The deflection speed of cyclic compression test is 2 mm min^-1^.

**Rheological test:** The rheological properties were measured by ARES-RFS rheometer. Each cylindrical sample tailored to 0.8 cm in diameter by 1 mm thickness was tested within the linear strain range (0.1%). The frequency sweep experiments were performed on the angular frequency ω range of 0.1-100 rad s^-1^ at 25^o^C for each polymer system.

**Crosslinking density:** The crosslinking density n was calculated via the formula: n = *E*_0_/(3RT), where *E*_0_ is the Young’s modulus, R the universal gas constant, and T the absolute temperature.

**Insoluble fraction:** The insoluble fraction was calculated via the formulas: Insoluble fraction = W_1_/W_0_ × 100%, where W_0_ is the original weight of the sample, W_1_ the weight of sample after being immerged into acetone for 72 h and dried under vacuum for 1 week. Insoluble fraction of PSeHCD elastomers represented the mass of the dried chemically crosslinked part after extraction of soluble fraction.

**Self-healing of PSeHCD elastomers:** For the self-healing of bulky materials, the strip was cut into two pieces, and then the two separate pieces were contacted with cross-sections and self-healed into an integrated whole under ambient conditions. The mechanical properties of healed materials were also tested. Scratch recovery tests were performed by scratching films with a blade. Then, the damaged film healed at room temperature. The change of the scratch-width was tracked using an optical microscope (Nikon, E100).

**Reprocessing of PSeHCD elastomers:** PSeHCD-72 elastomer was molded into one piece in a Teflon mold at 100^o^C for 10 min. Then the sample was reshaped into various shapes by different Teflon molds at 100^o^C, which was fixed while cooling to room temperature. The compression tests of original and reshaped samples were carried out at room temperature on an MTS insight mechanical analyzer.

**Functionalization of PSeHCD:** The PSeHCD-72 elastomer was shaped into a pony like sample. FITC/acetone solution (0.2 mg mL^-1^, 0.1 mL) was used to functionalize the surface. After solvent evaporation, the elastomer was put in to a drying oven at 40^o^C for 3 h and rinsed with distilled water for three times. Then, the elastomer was dried at 60^o^C under vacuum. A 365 nm UV flash light was used to excite fluorescence, which was observed by a digital camera.

***In vitro* degradation of PSeHCD elastomers:** An *in vitro* nonenzymatic degradation was carried out in phosphate buffered saline (PBS, pH=7.2-7.4). Every cylindrical sample (0.8 cm in diameter by 1 cm thickness) was weighed and put in 6 mL PBS solution. The incubation was performed in constant temperature at 37^o^C. In order to keep pH constant, the solution was changed every 7 days. At the predetermined harvest times, samples were washed by deionized water and dried in vacuum drying oven at 60^o^C. The degree of degradation was measured by the change of dry weight.

***In vitro*** **biocompatibility of PSeHCD elastomers testing:** PCL (Sigma-Aldrich, *Mw* = 14 kDa) solution (THF, 1 mg mL^-1^) was dropped onto the 24-well cell culture slides (Shanghai WoHong Biotechnology Co., Ltd., WHB-24-CS, 20 μL per slide). After the evaporation of the solvent, PCL covered 24-well culture cell slides were obtained. PSeHCD solution (THF, 1 mg mL^-1^) was dropped onto the 24-well cell culture slides (20 μL per slide). After the evaporation of the solvent, 24-well cell culture slides were transferred to a vacuum and heated at 120^o^C for 48 h to obtain PSeHCD elastomer covered 24-well cell culture slides. Passage 2 areolar fibroblasts originated from mouse tail dermal tissue were seeded on the pristine (blank), PCL covered, and PSeHCD elastomer covered cell culture slides placed in 24-well plates (1*10^4^ cells per well) for cytocompatibility evaluation. The CCK-8 (Dojindo Laboratories, Kumamoto, Japan) was used to analyze the cell viability on the day 1, 3, 5, 7 post-seeding. At predetermined time, 20 μL of CCK-8 was added to each well contained with 200 μL of MEM culture medium (11095-080, Gibco, ThermoFisher) and incubated at 37^o^C until color of medium become visible yellow before measuring the absorbance values at 450 nm via spectrophotometer (Synergy LX，BioTek，USA). Relative cell viability was calculated according to the optic density (OD) 450 values. Five specimens were used for each surface at one time point. Statistical analysis was performed using a one-way ANOVA test and a Tukey multiple comparison with a minimum confidence level of *p* < 0.05 was considered significant. All values are reported as a mean ± standard deviation.

**Supporting figures and tables**


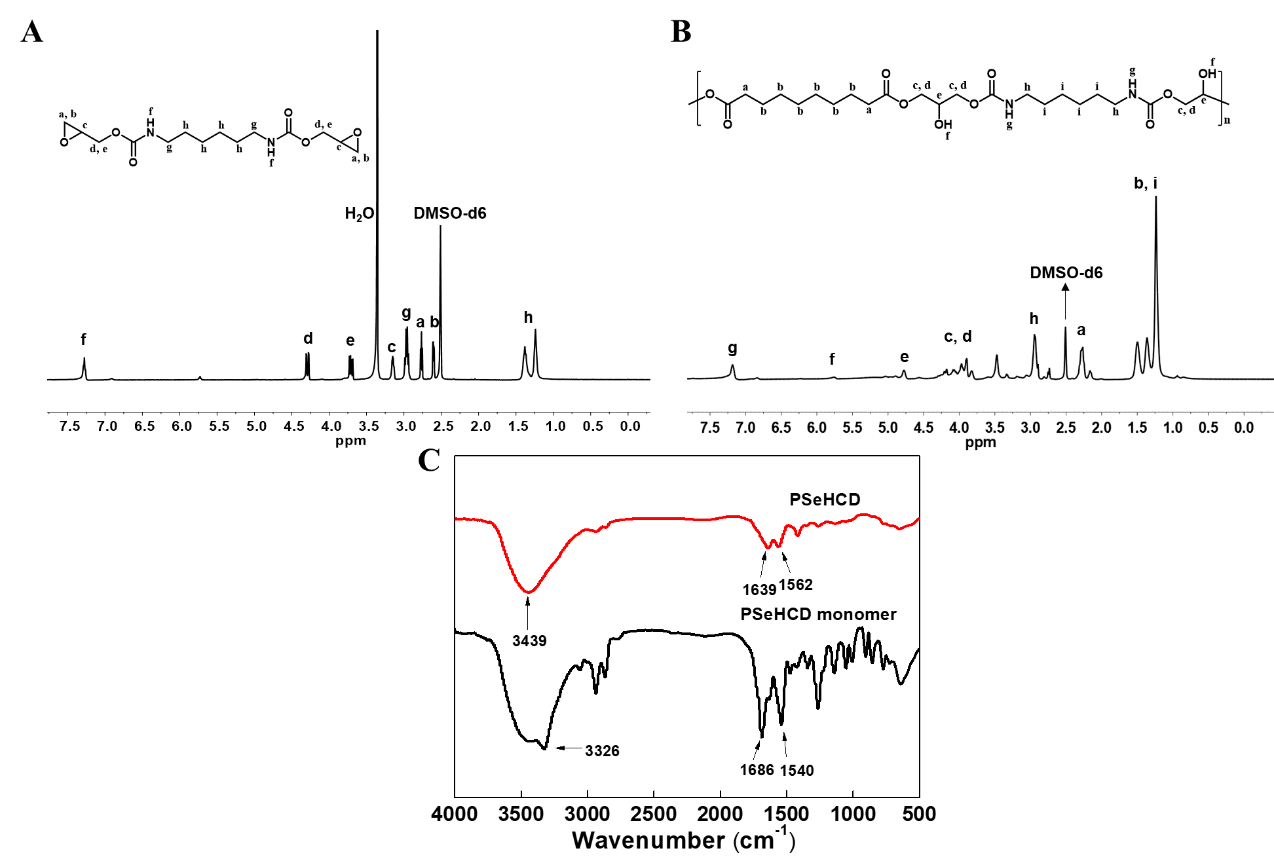


**Figure S1.** ^1^H NMR spectra of (A) monomer and (B) PSeHCD. (C) FTIR spectra of monomer and PSeHCD.


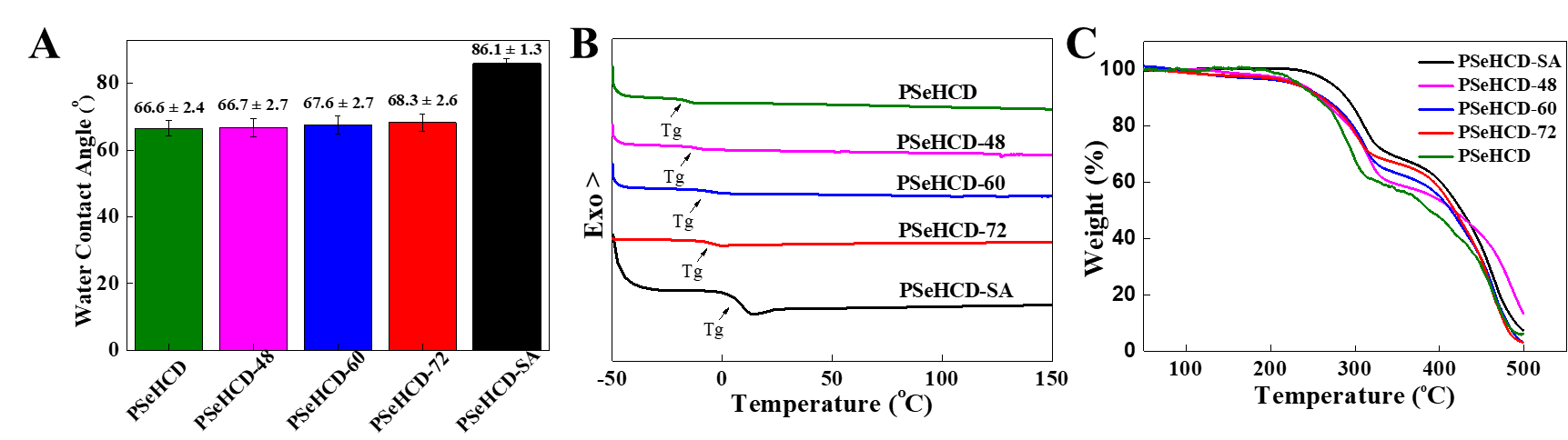


**Figure S2.** Physical properties of PSeHCD and the resultant elastomers. (A) The air-water contact angles. (B) DSC curves showed *T_g_* increased with the increase of crosslinking degree. No crystallization during this temperature range appeared. (C) TGA curves showed two-step thermal decomposition processes in all samples.

**Figure S3.** Storage modulus and loss modulus curves of PSeHCD-60 under temperature sweeping model.

**Table S1** Solubility of PSeHCD in common organic solvents (10 mg mL^-1^).

| Solvent^a^ | Hexane | Ethyl ether | THF | Ethanol | Ethyl acetate |
| --- | --- | --- | --- | --- | --- |
| Solubility^b^ | - | - | + | - | - |
| Solvent^a^ | Acetone | DMF | Methanol | Dimethyl sulfoxide | Water |
| Solubility^b^ | - | + | + | + | - |

^a^ Ranked by the polarity.

^b^ -, Insoluble, +, soluble.

**Table S2** Ratios of content of insoluble fraction in PSeHCD and resultant elastomers.

| Sample | PSeHCD | PSeHCD-48 | PSeHCD-60 | PSeHCD-72 | PSeHCD-96 | PSeHCD-SA |
| --- | --- | --- | --- | --- | --- | --- |
| Insoluble ratio^a^ (%) | 0 | 37.5±2.9 | 49.6±4.7 | 68.7±3.9 | 71.0±3.6 | 91.8±0.2 |

^a^All the experiments were carried out at room temperature.
